# Measuring and characterizing the quality of child care in Brazilian primary health care: a latent class analysis

**DOI:** 10.1590/0102-311XEN044025

**Published:** 2025-11-10

**Authors:** Maria del Pilar Flores-Quispe, Michelle Passos, Josemir Almeida, Ythalo Santos, Rosana Aquino, Anya Pimentel Gomes Fernandes Vieira-Meyer, Leandro da Luz, Eduarda dos Anjos, Acácia de Lima, Valentina Martufi, Naiá Ortelan, Maria Yury Ichihara, Mauricio Lima Barreto, Leila Amorim, Elzo Pereira Pinto

**Affiliations:** 1 Centro de Integração de Dados e Conhecimentos para Saúde, Fiocruz Bahia, Salvador, Brasil.; 2 Instituto de Saúde Coletiva, Universidade Federal da Bahia, Salvador, Brasil.; 3 Fiocruz Ceará, Eusébio, Brasil.; 4 Secretaria de Gestão do Trabalho e da Educação na Saúde, Ministério da Saúde, Brasília, Brasil.; 5 Instituto de Matemática e Estatística, Universidade Federal da Bahia, Salvador, Brasil.

**Keywords:** Primary Health Care, Child Health, Latent Class Analysis, Quality of Health Care, Health Services Research, Atenção Primária à Saúde, Saúde da Criança, Análise de Classes Latentes, Qualidade da Assistência à Saúde, Pesquisa sobre Serviços de Saúde, Atención Primaria de Salud, Salud Infantil, Análisis de Clases Latentes, Calidad de la Atención de Salud, Investigación sobre Servicios de Salud

## Abstract

This study aimed to characterize the quality of child health care and explore its relationship with municipal characteristics. Using data from the external assessment of the first cycle of the Brazilian National Program for Access and Quality Improvement in Primary Care (PMAQ-AB, acronym in Portuguese), this cross-sectional study evaluated 16,566 Family Health Strategy teams. In total, nine binary indicators were created based on recommendations from the Brazilian Ministry of Health. We employed latent class analysis and multinomial logistic regression to assess the quality of care and its association with region and the Brazilian Deprivation Index. Three patterns of quality of care were identified: high, intermediate, and low adequacy. The “high adequacy” pattern included 22.5% of teams, “intermediate adequacy” 60.2%, and “low adequacy” only 17.3%. Teams in the Northeast Region were over twice as likely to belong to the “high adequacy” pattern (OR = 2.34; 95%CI: 1.15-4.76) compared with those in the Central-West Region. For teams located in municipalities with moderate and low deprivation, the chance of belonging to the “high adequacy” pattern was 2.04 (95%CI: 1.44-2.89) and 9.08 (95%CI: 4.54-18.14) times higher, respectively, compared with the high deprivation municipalities. This study identified three patterns of quality of child care, with most teams characterized by an “intermediate adequacy” pattern. The quality of care was associated with the municipality’s characteristics. The methodology used in this study proved effective in characterizing the quality of care in a more consistent way.

## Abstract (pt)

Este estudo objetivou caracterizar a qualidade da assistência à saúde infantil e explorar sua relação com características municipais. Utilizando dados da avaliação externa do primeiro ciclo do Programa Nacional de Melhoria do Acesso e da Qualidade da Atenção Básica (PMAQ-AB), este estudo transversal avaliou 16.566 equipes da Estratégia Saúde da Família. Nove indicadores binários foram criados com base nas recomendações do Ministério da Saúde do Brasil. Utilizamos análise de classe latente e regressão logística multinomial para avaliar a qualidade da assistência e associações com região do país e com o Índice de Privação Brasileiro. Três padrões de qualidade da assistência foram identificados: alta, intermediária e baixa adequação. O padrão denominado “alta adequação” incluiu 22,5% das equipes, enquanto 60,2% das equipes foram categorizadas como “intermediária adequação” e apenas 17,3% estavam na categoria de “baixa adequação”. A chance de uma equipe pertencer ao padrão “alta adequação” foi mais de duas vezes maior (OR = 2,34; IC95%: 1,15-4,76) na Região Nordeste em comparação à Região Centro-oeste. Para equipes localizadas em municípios com privação moderada e baixa, a chance de pertencer ao padrão “alta adequação” foi de 2,04 (IC95%: 1,44-2,89) e 9,08 (IC95%: 4,54-18,14) vezes maior, respectivamente, em comparação aos municípios com alta privação. Este estudo identificou três padrões de qualidade do cuidado à criança. A maioria das equipes estava incluída no padrão “adequação intermediária”. A qualidade do cuidado estava associada com as características do município. A metodologia utilizada neste estudo permitiu caracterizar a qualidade do cuidado de forma mais consistente.

## Abstract (es)

El objetivo de este estudio fue caracterizar la calidad de la atención a la salud infantil y explorar su relación con las características municipales. Utilizando datos de la evaluación externa del primer ciclo del Programa Nacional de Mejoría del Acceso y Calidad de la Atención Básica (PMAQ-AB, por su sigla en portugués), este estudio transversal evaluó a 16.566 equipos de la Estrategia de Salud Familiar. Se crearon nueve indicadores binarios basados en las recomendaciones del Ministerio de Salud de Brasil. Utilizamos análisis de clases latentes y regresión logística multinomial para evaluar la calidad asistencial y su asociación con la región y el Índice de Privación Brasileño. Se identificaron tres patrones de calidad asistencial: alta, intermedia y baja adecuación. El patrón denominado “alta adecuación” incluyó al 22,5% de los equipos, el patrón “adecuación intermedia” agrupó al 60,2% de los equipos, y el patrón “baja adecuación” solo comprendió al 17,3%. La probabilidad de que un equipo perteneciera al patrón de “alta adecuación” fue más del doble (OR = 2,34; IC95%: 1,15-4,76) en la Región Noreste en comparación con la Región Centro-oeste. Para los equipos ubicados en municipios con privación moderada y baja, la probabilidad de pertenecer al patrón de “alta adecuación” fue 2,04 (IC95%: 1,44-2,89) y 9,08 (IC95%: 4,54-18,14) veces mayor, respectivamente, en comparación con los municipios de alta privación. Este estudio identificó tres patrones de calidad en la atención infantil. La mayoría de los equipos se incluyeron en el patrón de “adecuación intermedia”. La calidad asistencial mostró asociación con las características municipales. Nuestros resultados demuestran que la metodología empleada permitió caracterizar la calidad asistencial de manera más consistente.

## Background

Primary health care (PHC) is a cornerstone of health systems, delivering services ranging from health promotion and prevention to treatment and rehabilitation. It is crucial for fostering trust between health systems and communities ^1^. Despite commendable progress made by many countries in health indicators during the era of the Millennium Development Goals (MDGs), recent years have seen a growing concern about enhancing the quality of health care services.

The focus on quality improvement has been underscored by the World Health Organization (WHO), particularly in prioritizing efforts to reduce preventable child deaths ^2^. Recognizing the critical role of health systems in child health care, it is imperative to acknowledge that poor quality care can contribute to increased morbidity and mortality among children ^3^.

Measuring the quality of health services remains a challenge. First, defining the meaning of quality of care and what should be measured is important ^4^. Donabedian’s approach has been the most widely used in health systems studies ^5^. Thus, measuring the quality of structures, processes, and outcomes from the perspective of health professionals, managers, and patients is necessary ^5^. However, countries rarely conduct health facility surveys to collect data directly from health facilities or professionals at the national level. Overall, household surveys have been the main source of data to assess health service quality ^6^.

Facchini et al. defined quality of care as adherence to standards delineated in official documents and international guidelines for different age groups ^7^. In addition, the WHO emphasizes that health care must embody safety, effectiveness, timeliness, efficiency, equity, and a people-centered approach ^2^.

In Brazil, a notable expansion of PHC coverage, guided by the Family Health Strategy (FHS) − within the Brazilian Unified National Health System (SUS, acronym in Portuguese) − and its commitment to universal and comprehensive care, substantially increased health care access ^8^. Despite these advancements, the quality of care remains a considerable challenge within SUS.

The Brazilian National Program for Access and Quality Improvement in Primary Care (PMAQ-AB, acronym in Portuguese) was established in 2011 to address this challenge. This initiative aimed to enhance health care quality by providing financial incentives to municipalities based on external infrastructure and work process assessments ^9^. Coordinated by the Brazilian Ministry of Health, in conjunction with academic institutions, the assessment was conducted via interviews with municipal managers, health care professionals, and service users at the PHC facilities whose teams voluntarily decided to participate. The FHS teams received a score, and based on this, the Brazilian Ministry of Health provided financial incentives to the municipalities, which were used to provide salaries, training, technical assistance, supplies, infrastructure, and other resources that could aid improve the PHC quality ^10^.

In Brazil, standards for child health care provision within PHC services are guided by the *Cadernos de Atenção Básica nº 33* ^11^, which provides for service provision for fostering child growth and development, as well as promoting strategies for child care. Using PMAQ-AB data, previous studies had assessed the quality of child care via municipal aggregated (ecological) analyses ^12^ ^,^ ^13^, constructing a composite indicator ^14^ ^,^ ^15^, or estimating a composite index score ^16^ ^,^ ^17^ at the national or regional level.

Despite these efforts to assess the quality of child health care, the methods used did not enable a comprehensive characterization the quality of child care or measurement of the contribution of each indicator to identify strengths and weakness of PHC teams, nor for observing the differences in PHC performance. Furthermore, it is essential to analyze how municipal characteristics may influence care quality.

This study aims to characterize the quality of child health care provided by Brazilian PHC teams and explore its relationship with municipal characteristics.

## Methods 

### Study design and data

This cross-sectional study used data from the external assessment of the first cycle (2011-2012) of the PMAQ-AB, conducted by a national consortium of higher education institutions on behalf of the Brazilian Ministry of Health.

The evaluation instrument and logistics of the external assessment were developed under the coordination of the Brazilian Ministry of Health’s Department of Primary Care in partnership with Brazilian universities and other research institutions ^9^. This study used data from the following components of the survey instrument: Module I (observation of the structure of the PHC facility) and Module II (interview on PHC teams’ work processes). The dataset can be accessed at https://www.gov.br/saude/pt-br/composicao/saps/pmaq. Further information on the logistics of data collection is available at http://aps.saude.gov.br/ape/pmaq.

### Study population

In the first cycle of the PMAQ-AB, 17,202 PHC teams participated in the external assessment (by voluntary adherence), representing 51% of PHC teams of Brazil. These teams were distributed across 3,935 municipalities (70.7%) ^18^. In Brazilian primary care, there are different modalities of health teams. The Family Health Strategy (FHS) is the main model of primary care organization in Brazil and serves as the gatekeeper to the national health system. The FHS is characterized by providing comprehensive and universal care to a defined geographical catchment area (up to 4,000 inhabitants) ^10^. Considering the alignment of this modality of PHC teams with the recommended PHC principles for the Brazilian context, we included 16,566 PHC teams from the FHS, corresponding to 49.6% of all active FHS teams in 2012, distributed across 69.3% of Brazilian municipalities.

### Indicators of the quality of child health care and covariates

Following the Brazilian *Cadernos de Atenção Básica nº 33* ^11^ for child care from the Brazilian Ministry of Health ^11^, 20 yes/no questions were selected from the PMAQ-AB external assessment instrument. These were used to construct eight indicators: postnatal care, record-keeping practices, traditional surveillance activities, surveillance of external causes, community outreach, breastfeeding guidance, care provision, and child care program offer. For each of these eight indicators to be considered “adequate”, they had to comply with standards established for child care in PHC by the Brazilian Ministry of Health. In addition, a list of medicines for child care was selected according to WHO and Brazilian lists of essential medicines ^19^ ^,^ ^20^ ^,^ ^21^. These medicines were arranged into five groups: antiparasitic drugs; vitamins, multivitamins and oral rehydration salts; anti-asthmatic agents; analgesics and antipyretics; and antibacterials. To consider this indicator “adequate”, the PHC facility had to supply of at least one medicine from each group. An indicator of medicines was then defined. In total, nine binary indicators (adequate/inadequate) were constructed to characterize the quality of child care, as detailed in Table 1. 

**Table:** 1 Indicators, variables, frequency of variables, definition and frequency of adequacy of indicators of quality of child health care. Brazilian National Program for Access and Quality Improvement in Primary Care (PMAQ-AB, acronym in Portuguese), Cycle I, 2012.

Indicator	Items	% yes	Adequacy definition and frequency (%)
Postnatal care	In order to guarantee a postnatal visit within 10 days after delivery, a Comprehensive Healthcare Week carries out home visits to enroll women in the service	90.2		The PHC team performs the three strategies to guarantee a postnatal visit within 10 days after delivery and provides special consultation time and postnatal visits by a physician or nurse **Adequate:** affirmative responses to all five activities (30.2)
	In order to guarantee a postnatal visit within 10 days after delivery, a member of the team carries out home visits to enroll women in the service	82.7		
	In order to guarantee a postnatal visit within 10 days after delivery, the team provides special consultation time for any day of the week	53.1		
Medicines	A physician carries out postnatal visits	67.3		This indicator was composed of five groups of essential medicines for the care of children. For each group. the PHC facility had to have at least one medicine **Adequate:** the PHC facility always has at least one medicine for each medicine category (65.2)
	A nurse carries out postnatal visits	89.8		
	Antiparasitic	72.5		
	Vitamins, multivitamins, oral rehydration salts	79.6		
	Anti-asthmatic agents	73.3		
	Analgesics and antipyretics	76.4		
	Antibacterials	76.2		
Record keeping practices	The team uses the child care booklet to monitor children	94.6		The PHC team uses the child care booklet to monitor the children, and there is a copy/record of the child care booklet or another form with equivalent information in the PHC facility **Adequate:** affirmative responses to both questions (76.8)
	There is a copy/record of the child care booklet, or another form with equivalent information in the PHC facility	79.2		
Surveillance − traditional activities	Records of children in the territory with regards to growth and development	89.3		The PHC team has records of children regarding growth and development, nutritional status, and Guthrie test screening. Additionally, the PHC team has an updated record of children up to two years old in the territory, as proven by showing a document **Adequate:** affirmative responses to all four questions (56.9)
	Records of children in the territory with regards to nutritional status	86.1		
	Records of children in the territory with regards to Guthrie test screening	78.6		
	The PHC team has updated records of children up to two years old in the territory. There is a document to prove it	74.5		
Surveillance − external causes	Records of children in the territory with regards to accidents	33.2		The PHC team has records of children in the territory regarding accidents and domestic violence **Adequate:** affirmative responses to both questions (28.3)
	Records of children in the territory with regards to domestic violence	34.0		
Community outreach	The PHC team conducts community outreach to identify children born with low birthweight	87.7		The PHC team conducts community outreach to identify children born with low birth weight, prematurely, and those behind on their growth and development follow-up **Adequate:** affirmative responses to all three activities (68.4)
	The PHC team conducts community outreach to identify children born prematurely	81.0		
	The PHC team conducts community outreach to identify children who are behind on their growth and development follow-up	77.4		
Breastfeeding	The PHC team offers health education and health promotion activities for pregnant women and women in postpartum (breastfeeding guidance)	87.4		The PHC team offers health education and promotion activities about breastfeeding for pregnant women and women in postpartum **Adequate:** Affirmative responses to the question (87.4)
Care provision	The PHC team has defined protocols and therapeutic guidelines for: children under two years old (growth/development)	66.5		The PHC team has defined protocols and therapeutic guidelines for children under two years old (growth/development) and carries out growth and development follow-up for children up to two years old **Adequate:** affirmative responses for both activities (60.0)
	The PHC team conducts growth and development follow-up for children up to two years old	85.4		
Program offer	Child growth and development follow up is among the services provided to special interest groups	78.3		Child growth and development follow-up is among the services provided by the PHC team **Adequate:** affirmative response for the activity. (78.3)

PHC: primary health care.

In total, two municipal level variables were used as covariates to describe their relationships with the quality of child health care: geographic region and the Brazilian Deprivation Index (IBP, acronym in Portuguese). The Brazilian regions are North, Northeast, South, Southeast, and Central-West. The IBP was created using data from the 2010 Brazilian Population Census and combined three indicators: percentage of households with per capita income below half the minimum wage; percentage of illiterate people aged seven or older; and percentage of people with inadequate access to sewage, water, garbage collection, and without an indoor toilet or bath/shower. The composite measure was divided into quintiles and classified from least to most deprived. Allik et al. ^22^ describe the details of the IBP’s construction. For this study, the IBP was categorized as low deprivation (first and second quintiles), moderate deprivation (third quintile) and high deprivation (fourth and fifth quintiles). 

### Statistical analysis

All variables in this study were categorical. Thus, frequencies were used to describe the nine indicators of child health care quality and the covariates (region and IBP). Latent class analysis (LCA) was adopted to characterize child care quality based on the nine PMAQ-AB binary indicators. LCA is commonly used when a categorical variable of interest is not measured directly or without error, referred to as a latent variable, and the indicators are categorical. In this study, the latent variable identified patterns of quality of child care. The method evaluates response patterns from the observed categorical indicators to identify and characterize latent classes representing the concept of interest (i.e., quality of child health care). The results of LCA are described based on two sets of parameters: the probability of membership in each latent class (or class prevalence) and the probability of a given response for the observed variable, conditional on class membership ^23^. The number of classes for the model was selected using the Akaike information criterion (AIC), the Bayesian information criterion (BIC), and the Vuong-Lo-Mendell-Rubin likelihood ratio test (which compares the model with K classes and the model with K-1 classes). Entropy was used to describe classification error, with higher entropy values indicating greater precision in group membership classification and clearer distinction between latent classes. Bivariate residuals from LCA fit were analyzed to evaluate the local independence assumption.

**Figure 1:**
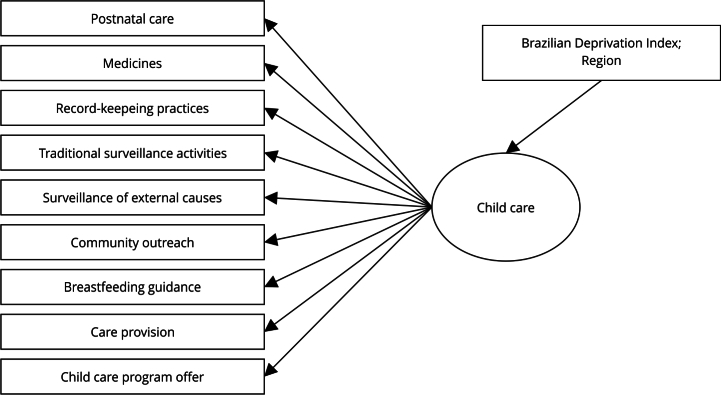
Latent classes analysis (LCA) model for quality of child health care in primary health care teams: indicators, latent variable and associated determinants.

An LCA with covariates was then used to assess associations between latent class membership (quality of child care) and the covariates (region and IBP) (Figure 1). This methodology employs multinomial logistic regression, in which the outcome is the latent variable, providing odds ratio (OR) estimates and 95% confidence intervals (95%CI). In addition, all analyses corrected standard errors for the non-independence of observations due to cluster sampling ^24^, with clusters defined at the municipality level, as multiple health teams may be located in the same municipality. Moreover, the LCA residual association model was used to address violations of the local independence assumption ^25^. Parameter estimation for the LCA with covariates was performed using a three-step approach, which includes a simple correction for a common source of bias ^26^, known as the BCH ,(Bolck-Croon-Hagenaars) method. 

LCA is used in contexts in which multiple observed binary indicators reflect an underlying, unobservable dimension. In this study, it was employed to characterize and describe distinct team profiles, revealing the underlying heterogeneity in the quality of child care.

Analyses were performed using Stata version 17.0 (https://www.stata.com) and Mplus version 8.6 (https://www.statmodel.com/).

### Ethical consideration

This study used public data from the PMAQ-AB external assessment; therefore, according to Brazilian regulations, no ethical approval was necessary. Moreover, the first cycle of the PMAQ-AB protocol was approved at the national level by the Federal University of Pelotas (code 38/2012).

## Results

Table 1 shows the percentage of adequacy of PHC teams for the child health care indicators. The indicator with the highest adequacy was the provision of health education and promotion activities related to breastfeeding for pregnant and postpartum women (87.4%). In contrast, the indicators with the lowest adequacy were PHC teams performing actions to ensure a postnatal visit within 10 days after delivery (30.2%) and maintaining records for children in the territory related to accidents and domestic violence (28.3%). 

Regarding the covariates, 38% of PHC teams were located in municipalities in the Southeast, 32.1% in the Northeast, 17.2% in the South, 6.4% in the Central-West, and 6% in the North regions. In relation to the IBP, 54.7% of PHC teams were in municipalities with high deprivation, whereas 16.7% and 28.6% were in municipalities with moderate and low deprivation, respectively. We highlight that this distribution reflects the voluntary participation of PHC teams in the external assessment during the first cycle of the PMAQ-AB. 

LCA models with one, two, three, and four latent classes were adjusted, and their goodness of fit measures were compared (Table 2). The AIC and BIC values for the model with three classes were 31,193.2 and 31,301.2, respectively, and the entropy value was 0.535. Based on these results, as well as better interpretability in highlighting indicators of child care quality, the three-class model was selected.

**Table 2:** Goodness of fit measures varying the number of latent classes for the quality of child health care. Brazilian National Program for Access and Quality Improvement in Primary Care (PMAQ-AB, acronym in Portuguese), Cycle I, 2012.

	Number of classes			
	1	2	3	4
Number of estimated parameters	-	7	14	21
AIC	94084.9	20795.6	31193.2	39022.3
BIC	94177.5	20849.7	31301.2	39184.3
Entropy	-	0.529	0.535	0.509
		1 vs. 2 classes	2 vs. 3 classes	3 vs. 4 classes
Vuong-Lo-Mendell-Rubin likelihood ratio test	-	0.451	0.368	0.600

AIC: Akaike information criterion; BIC: Bayesian information criterion.

Table 3 shows the class prevalence and conditional probabilities for the three empirically identified patterns (latent classes). The pattern labeled “high adequacy” included 22.5% of PHC teams, characterized by higher conditional probabilities for seven indicators (greater than 70%) compared to the other PHC teams. Only the indicators surveillance of external cause and postnatal care had conditional probabilities lower than 70% (62.1% and 49.9%, respectively) in this class. The pattern called “intermediate adequacy” involved 60.2% of PHC teams and had the highest probabilities for adequate breastfeeding guidance (87.8%) and child care program offer (81.9%), and the lowest probability for surveillance of external causes (20%). The third pattern, “low adequacy”, included 17.3% of PHC teams and was characterized by low probabilities for all indicators, except for breastfeeding guidance (70.7%). This pattern had the lowest probabilities for both surveillance indicators: traditional surveillance activities (10%) and surveillance of external causes (3.8%). 

**Table 3:** Characterization of child health care quality pattens via latent class analysis for primary health care teams. Brazilian National Program for Access and Quality Improvement in Primary Care (PMAQ-AB, acronym in Portuguese), Cycle I, 2012.

	Child health care quality patterns (%)		
	High adequacy	Intermediate adequacy	Low adequacy
Prevalence	22.5	60.2	17.3
Indicators	Conditional probabilities		
Postnatal care	49.9	27.0	11.2
Medicines	74.1	64.6	54.5
Record-keeping practices	93.0	77.9	50.5
Traditional surveillance activities	91.2	56.2	10.0
Surveillance of external causes	62.1	20.0	3.8
Community outreach	96.6	67.5	30.8
Breastfeeding guidance	98.4	87.8	70.7
Care provision	88.9	60.7	16.9
Child care program offer	95.9	81.9	43.0

Table 4 shows the estimates for the relationships (OR and 95%CI) between municipality characteristics and the latent patterns of child care quality. The pattern “low adequacy” was the reference latent class. In the adjusted analysis, the chance of belonging to the “high adequacy” pattern for PHC teams in the Northeast Region was more than twice (OR = 2.34; 95%CI: 1.15-4.76) that of those from the Central-West Region. Regarding the IBP, when PHC teams were located in municipalities with moderate and low deprivation, the chance of belonging to the “high adequacy” pattern was 2.04 (95%CI: 1.44-2.89) and 9.08 (95%CI: 4.54-18.14) times, respectively, compared to PHC teams in municipalities with high deprivation. Moreover, a similar result was observed for the “intermediate adequacy” pattern. PHC teams in the Northeast Region had more than twice the chance of belonging to the “intermediate adequacy” pattern compared to PHC teams in the Central-West Region (OR = 2.77; 95%CI: 1.84-4.17). When PHC teams were in municipalities with moderate and low deprivation, the chance of belonging to the “intermediate adequacy” pattern was 1.67 (95%CI: 1.26-2.22) and 2.79 (95%CI: 1.91-4.09) times, respectively, compared to those in municipalities with high deprivation.

**Table 4:** Estimates of association between municipality characteristics and latent patterns of quality of child health care in primary health care teams. Brazilian National Program for Access and Quality Improvement in Primary Care (PMAQ-AB, acronym in Portuguese), Cycle I, 2012.

Covariates *	Patterns of quality of child health care **			
	High adequacy		Intermediate adequacy	
	Adjusted OR	95%CI	Adjusted OR	95%CI
Region				
North	1.27	0.61-2.65	0.99	0.61-1.62
Northeast	2.34	1.15-4.76	2.77	1.84-4.17
South	1.04	0.48-2.25	0.96	0.61-1.51
Southeast	1.69	0.78-3.69	1.36	0.88-2.12
Brazilian Deprivation Index				
Moderate deprivation	2.04	1.44-2.89	1.67	1.26-2.22
Low deprivation	9.08	4.54-18.14	2.79	1.91-4.09

95%CI: 95% confidence interval; OR: odds ratio.

* Reference categories for covariates: Central-West Region and municipalities with high deprivation;

** The “low adequacy” pattern is the reference in the latent class analysis with covariates.

## Discussion

This study proposed a pragmatic way to measure the quality of child care, characterizing PHC teams according to their performance on a set of nine indicators developed following Brazilian Ministry of Health guidelines for the care of children in primary health care. We highlight the use of LCA in this study to characterize child care quality, as it enabled us to distinguish three patterns of care quality and identify the contribution of each indicator to the respective pattern, demonstrating weaknesses in the compliance with interventions needed to deliver a high quality of care. 

Our findings identified three patterns of child health care quality among PHC teams: high adequacy, intermediate adequacy, and low adequacy. More than half of the PHC teams were characterized as having intermediate or low adequacy. Moreover, the municipality’s geographical region and the IBP were associated with PHC team adequacy patterns.

The analysis of child health care quality across regions showed that PHC teams in the Northeast Region were more likely to be characterized by high or intermediate adequacy. Despite historically facing socioeconomic disadvantages, barriers to accessing health services, poorer health indicators, and greater inequalities, the Northeast has demonstrated substantial improvements in health indicators over time. This progress is likely associated with the implementation of national programs that primarily focused on the Northeast and North regions. 

An early example is the Community Health Workers Program (PACS, acronym in Portuguese), created in 1991 to improve maternal and child mortality indicators, initially in the Northeast Region ^27^. In 2002, the Brazilian Ministry of Health estimated that the highest concentration of active community health workers (CHW) was in the Northeast ^28^. Building on the experience with PACS, the Ministry recognized the importance of focusing on the family − rather than only the individual − as the unit of health intervention, leading to the creation of the Family Health Program (PSF, acronym in Portuguese) in 1994. From 1999 to 2004, the Northeast Region had the greatest increase in PSF coverage as shown by Facchini et al. ^29^. Similarly, from 2006 to 2012, this region had the highest coverage levels of the FHS ^30^. Dourado et al. ^31^ demonstrated an association between more consolidated FHS implementation and the presence of a usual source of care, particularly in the Northeast, North, and Central-West regions. The FHS, the main organizational model of PHC in Brazil, has played a key role in expanding access, organizing and coordinating care, and promoting health equity. Notable results of these implementations include reductions in under-5 mortality rates ^32^ ^,^ ^33^ ^,^ ^34^, increased antenatal care indicators ^35^, and better performance on prenatal care quality indicators in the Northeast Region ^36^. 

The IBP was also an important factor for PHC teams belonging to the high or intermediate adequacy patterns, with the greatest likelihood among teams from municipalities with low deprivation. The socioeconomic context of a municipality, region, or country is associated with health indicators. A study using the IBP also observed lower mortality and hospitalization rates due to primary care sensitive conditions in municipalities with low deprivation ^37^.

Studies in low- and middle-income countries have shown that the wealthier deciles or quintiles had the highest rates of skilled attendance at delivery ^38^ and skilled antenatal and postnatal care ^39^. This may be because municipalities, regions, or countries with better financial resources are able to provide stronger infrastructure for health services and have greater economic capacity to hire more health professionals. 

Regarding child care quality, most studies employ the PCATool child version. Results have varied greatly, and these studies are generally conducted in specific municipalities or regions. The attribute continuity of care seemed to have the highest score, and PHC teams in services with the FHS model in Brazil performed better than those in the traditional model ^40^ ^,^ ^41^. The PCATool child version is used to assess care for children of all ages, typically aged from 0 to 14 years. In our study, we did not employ the PCATool, and instead, information on child care was provided by a health professional who is part of the PHC team. Despite this, our approach offers a comprehensive picture of quality performance based on both normative standards and the Donabedian’s approach. In addition, the LCA method enabled the identification of different classes and the performance of each indicator within them.

Studies using national PMAQ-AB data have employed different methods to measure child care quality. Vieira-Meyer et al. ^16^ constructed a composite index with nine questions on the child care process and obtained a score over 0.70 (range of 0 to 1.0) in 2012, suggesting good quality of care. Another study, also using work process information, found that 63.5% of PHC teams had a high level of quality in child health care, and strong coordination among PHC teams was associated with better quality of care ^42^. These studies, which used information similar to ours, produced results in the same direction, demonstrating the efforts of PHC teams to achieve good quality of child care. 

Both international and national studies, using different datasets and methodologies, converge on the need for strategies to improve health care quality, with a particular emphasis on child care, as this population requires continuous care. Additionally, there is a clear need for quality monitoring systems, with appropriately gathered information directly from health services and professionals to assess structure and work processes. This collection should be conducted periodically with a standardized instrument to enable effective monitoring over time. An example is the PMAQ-AB itself, which − with its significant amount of data − enabled a robust analysis such as the LCA used in this study, identifying indicators that still need to improve their level of adequacy, for example, in intermediate adequacy teams, which was the majority in the first cycle of the Program.

In this study, the breastfeeding indicator had the highest frequency of adequacy compared to the other eight indicators, and the highest conditional probability across all three adequacy patterns. This finding demonstrates PHC teams strong efforts to promote breastfeeding during prenatal and postpartum periods. Systematic reviews have shown that educational and structured breastfeeding programs at the primary care level during these periods significantly improve breastfeeding practices in both the short and long term ^43^ ^,^ ^44^. In the Brazilian PHC context, successful initiatives such as the Brazilian Breastfeeding Network may explain the good performance of this indicator ^45^ ^,^ ^46^. 

In contrast, postnatal care had only 30.2% adequacy and the lowest conditional probability in the “high adequacy” pattern. Despite this low performance, Brazil has normative documents and policies that guide interventions in this period, highlighting the importance of the First Comprehensive Healthcare Week ^47^. During this period, maternal and neonatal morbidity and mortality can be prevented. The mother needs a physiological and clinical-gynecological assessment, an investigation of difficulties with breastfeeding, and an evaluation of her psycho-emotional condition ^48^. The baby should undergo a complete physical examination. Weight and height should be measured; the umbilical cord should be examined for possible infection; breastfeeding technique should be observed; advice should be given on the supine position for sleeping to prevent sudden infant death; and the heel prick test, as recommended by the Brazilian National Newborn Screening Program (PNTN, acronym in Portuguese), should be conducted to detect any conditions that, with timely treatment, could improve the baby’s health ^11^ ^,^ ^14^. 

Two studies showed that postpartum consultations remained at around 50% or less, with the Central-West Region showing the lowest adequacy ^18^ ^,^ ^49^. An explanation may be that this region has lower FHS coverage compared to the national average, also seen in the Southeast ^31^. The expansion the FHS model could increase the provision of actions and services ^7^. A characteristic of the FHS is the composition of a minimum team (a physician, nurse, nursing technician, and two or more CHWs) that can provide comprehensive care. CHWs in particular play an important role in ensuring postpartum consultations, as shown in a study in Pernambuco State where only 42.1% of women received a home visit within the first week after hospital discharge, most often performed by a CHWs (46.2%) ^50^. 

The indicator for surveillance of external causes had the lowest adequacy (28.3%) and the lowest conditional probabilities in the intermediate and low adequacy patterns. This indicator refers to PHC teams keeping records of children in their territory regarding accidents and domestic violence. According to the Brazilian National Policy for Comprehensive Child Health Care, violence and accidents are classified as external causes and represent a serious public health issue ^51^. Exposure to violence − physical or psychological − is a risk factor for child development, especially in the first years of life ^52^. Likewise, inadequate child care can increase the risk of accidents, some of which may result in death ^52^. 

A UNICEF Brazil report indicated that from 2016 to 2020, of 34,918 intentional violent deaths among children and adolescents, 1,070 were children under the age of nine, and 40% of these occurred at home. The report also highlighted a 27% increase in annual violent deaths among children aged 0 to 4, which may reflect domestic violence ^53^. 

Surveillance of external causes does not appear to have been established as a routine activity, unlike surveillance of traditional indicators such as child growth, development, and nutritional status. This may have occurred because the latter became global priorities due to childhood malnutrition being recognized as a major health issue. 

However, the WHO highlights the need for training health professionals to screen, prevent, and manage cases of violence or accidents involving children ^2^. In Brazil, *Cadernos de Atenção Básica n* ^*o*^ *33* for child health care also highlights that PHC professionals have close contact with communities − particularly children and their families − and that home visits represent a unique opportunity to conduct educational activities on accident prevention, identify and monitor children and families in situations of violence, and develop strategies for continuity of care ^11^.

The identification of low performance in certain indicators of child health care − such as postnatal care and surveillance of external causes − highlights opportunities for targeted interventions aimed at improving these essential services. Efforts should also focus on strengthening the work of PHC teams − whether or not they are part of the FHS − recognizing their central role in delivering comprehensive and equitable care. Furthermore, the observed association between higher adequacy levels in child health care indicators and better socioeconomic conditions underscores the importance of directing policies and investments toward more vulnerable contexts to promote equity in the quality of health services.

This study has some limitations. First, the results should not be generalized to all Brazilian PHC teams because participation in the PMAQ-AB was voluntary, introducing selection bias, particularly in the first cycle of the Program, when only slightly more than 50% of teams were evaluated. Furthermore, participating teams belonged to municipalities whose managers had voluntarily decided to participate, which suggests that these teams were already higher performing and that their facilities may have had better infrastructure to achieve good ratings and benefit from incentives offered by the Program. This limitation could have led to overestimation of quality. However, we found that the largest proportion of teams fell into the “intermediate” quality pattern. Information bias may also have impacted results. Nonetheless, in constructing indicators, whenever the PMAQ-AB instrument included a complementary question asking whether documentation was available to verify an action, this information was incorporated to classify it as adequate. In addition, the medicines indicator was constructed based on Module 1 data gathered via direct observation by a trained interviewer. 

The study’s strengths include the use of a large nationwide sample. In the first PMAQ-AB cycle, the PHC teams included in this study represented 49.6% of all teams active in Brazil in 2012. Another strength is the data collection, which combined direct observation of the PHC facilities and interviews with health professionals. The constructed indicators were based on national guidelines and standards for child care, mainly from the Brazilian Ministry of Health. Additionally, the methodological strategy adopted to describe child care quality in primary care in Brazil incorporated potential measurement errors by employing LCA with covariates, along with corrections for: (i) violations of local independence assumptions, using the approach proposed by Visser et al. ^25^, and (ii) the correlated data structure (multiple health teams in the same municipality). Thus, the estimates from these analyses are prone to be more robust.

## Conclusion

Our findings identified three patterns of quality of care for children, as well as the contribution of each indicator included in the model, showing that latent class analysis enabled the identification of both strengths and weaknesses in complying with the strategies recommended by the Brazilian Ministry of Health for child care in PHC. Likewise, our study demonstrated that region and the IBP are associated with teams being characterized by patterns of high, intermediate, or low adequacy. This highlights the need, at national, regional, and municipal levels, to reduce inequalities in the provision of child care within PHC. Policy implementation should consider these discrepancies and formulate strategies and actions to mitigate them.

## Data Availability

The databases used in the study, including extraction codes, analyses, and results, are available in the repository: https://github.com/cidacslab/cidacs-phc/tree/be454ff81b3d7afa07dcf5f341ff2ceca0fe3390/Latent%20Class%20Analysis_Child%20Care_GITHUB. The sources of information used in the study are indicated in the body of the article.
